# Effects of di-(2-ethylhexyl) phthalate on growth, metabolism, and virulence of the plant pathogenic bacterium *Acidovorax citrulli*

**DOI:** 10.3389/fcimb.2023.1228713

**Published:** 2023-08-25

**Authors:** Yu-Ri Kim, Mee Kyung Sang

**Affiliations:** Division of Agricultural Microbiology, National Institute of Agricultural Sciences, Rural Development Administration, Wanju, Republic of Korea

**Keywords:** di-(2-ethylhexyl) phthalate (DEHP), phthalate, *Acidovorax citrulli*, metabolism, virulence

## Abstract

*Acidovorax citrulli* is a seed-borne bacterial pathogen that causes bacterial fruit blotch in cucurbits and severely affects the production of cucumbers and watermelons globally. In this study, we investigated the effects of di-(2-ethylhexyl) phthalate (DEHP) on the growth, metabolism, and virulence of *A. citrulli*. Bacterial population was not affected by DEHP exposure; moreover, significant changes were not observed in lipid peroxidation, membrane permeability, and nucleic acid leakage. However, palmitoleic acid content was increased in the cell membrane of DEHP-exposed *A. citrulli*. Further, DEHP exposure increased the activity of TCA cycle-related enzymes, including α-ketoglutarate dehydrogenase and succinyl-CoA synthetase, along with increase in the content of glutamate, succinate, fumarate, and malate in TCA cycle. Additionally, total 270 genes were differentially expressed by the treatment, of which 28 genes were upregulated and 242 genes, including those related to translation, flagellum-dependent cell motility, and flagellum assembly, were downregulated. Regarding virulence traits, swimming activity was decreased in DEHP-exposed *A. citrulli*; however, biofilm formation was not affected in *in vitro* assay. Moreover, relative expression of pathogenicity genes, including *hrpX* and *hrpG*, were decreased in DEHP-exposed *A. citrulli* compared to that of unexposed *A. citrulli*. Therefore, these results suggest that DEHP accumulation in soil could potentially influence the metabolism and virulence traits of *A. citrulli*.

## Introduction

Phthalates are a group of chemical compounds that are commonly used as plasticizers for flexibility and durability in various other products, including adhesives, sealants, and personal care products (Gao and Wen, 2016). Phthalates are toxic to the environment because they are non-biodegradable and persist in the environment for a long time. Owing to their extensive presence, they can accumulate in the food chain and cause potentially harmful effects on wildlife and humans (He et al., 2013; Groh et al., 2019; Li et al., 2021). Studies have shown that phthalates can disrupt hormone function in both animals and humans, leading to developmental and reproductive problems. For example, exposure to phthalates during pregnancy is associated with various adverse effects on fetal development, including low birth weight, developmental delays, and changes in genital development. In addition to their effects on human health, phthalates have negative impact on the environment (Groh et al., 2019). They can leach from plastic products, contaminate soil and water, and have been found at high levels in some aquatic ecosystems (Li et al., 2021). Studies have shown that phthalates affect the growth and survival of certain organisms (Liu et al., 2009; Kim et al., 2019; Li et al., 2021). Owing to their potentially harmful effects on human health and environment, some phthalates have been restricted or banned in certain countries. However, they are still widely used in many products globally.

Di-(2-ethylhexyl) phthalate (DEHP) is commonly used in various products, including vinyl flooring, medical devices, food packaging, and toys. However, DEHP has been found to have toxic effects on both humans and environment. Studies have shown that DEHP exposure can cause reproductive and developmental problems as well as liver and kidney damage. In addition, DEHP has a negative impact on the environment (Chen et al., 2011; Shen et al., 2015) because it is non-biodegradable and can persist in the environment for a long time. Additionally, it can contaminate soil and water, and has been found at high levels in some ecosystems.

Furthermore, DEHP can negatively affect soil biology (Wang et al., 2017; Gao et al., 2020b). When it is released into the soil, it can remain and accumulate the soil over long time, leading to potentially harmful effects on soil microorganisms and other biota (Jiang et al., 2022). Studies have shown that DEHP can affect the soil microbial activity, diversity, and composition of soil microbial community (Gao et al., 2020a). For example, Wang et al. (2009) found that DEHP exposure led to a decrease in soil microbial biomass and activity as well as changes in the relative abundance of certain bacterial groups; Gao et al. (2020b) suggested that DEHP could potentially affect soil nutrient cycling and plant growth, thereby indirectly affecting the soil biota. Additionally, DEHP exposure reduced soil nitrogen availability, leading to decreased plant growth and biomass (Tao et al., 2021). These studies demonstrate that DEHP exposure can have significant effects on soil bacterial biology, potentially altering soil nutrient cycling and affecting plant growth and health.

*Acidovorax citrulli* is a gram-negative bacterium that causes bacterial fruit blotch (BFB) disease in cucurbit plants such as watermelon, cantaloupe, and cucumber (Burdman and Walcott, 2012). This disease is frequently outbreaks around the world, resulting in significant economic loss by reducing crop yields, decreasing fruit quality, and increasing postharvest loss (Burdman and Walcott, 2012; Zhao et al., 2020). BFB is characterized by water-soaked lesions on fruits and leaves, which can progress into brown, necrotic areas (Burdman and Walcott, 2012). This bacterium can also infect seeds, leading to seedling rot, and eventually plant death. Infected fruits become inedible because of the presence of bacteria and the development of secondary fungal infections. *A. citrulli* possesses several virulence factors, including exopolysaccharides (EPS), which are sticky, carbohydrate-based substances that enable it to adhere to the plant, colonize host plant tissues, and cause disease in host plants (Burdman and Walcott, 2012). EPS also protects the bacterium from the host plant immune system and helps it form biofilm on plant surfaces, which can increase its resistance to environmental stressors. The type III secretion system (T3SS), a specialized protein secretion system, is used by the bacterium to inject effector proteins into the host plant cells. These effector proteins can manipulate the host cell signaling pathways, suppress the host plant immune response, and enable bacteria to extract nutrients from the host. Moreover, *A. citrulli* produces extracellular enzymes, such as pectinases and cellulases, which allow the bacterium to break down plant cell wall components and access nutrients. Additionally, the bacterium can produce siderophores, specialized molecules that enable it to scavenge iron from the host plant. These virulence factors enable *A. citrulli* to colonize and cause disease in the host plants, making it an important pathogen in cucurbit crops.

There is currently no evidence of a direct relationship between DEHP and phytopathogenic bacteria. However, DEHP persists in soils, and exposure may affect phytopathogens, including their population, metabolism, and virulence. Therefore, this study was aimed at evaluating the toxic effects of DEHP to *A. citrulli*, exploring its effects on metabolism and virulence of the bacterium, and investigating changes in gene expression following DEHP exposure. Understanding the potential impact of DEHP exposure on the seed-borne plant pathogenic bacteria *A. citrulli* is important for assessing the risk and control of BFB in agriculture.

## Materials and methods

### Bacterial strain preparation

The bacterial strain *Acidovorax citrulli* (KACC no. 17002) used in this study was provided by the Korean Agricultural Culture Collection. To prepare the bacterial suspension, *A. citrulli* was grown on nutrient agar (NA, Difco, Detroit, MI, USA) at 28°C for 48 h, followed by culture of single colony in nutrient broth (NB) at 28°C for 48 h.

### Bacterial culture and population in a medium containing di-(2-ethylhexyl) phthalate

The DEHP (200 mg/10 mL in 80% acetone, as a stock) was amended with diluted NB (1:10) media at 0.5% of the final volume. *A. citrulli* (3.6 × 10^8^ colony forming units (CFU)/mL, 1 mL) was grown in 100 mL of diluted NB supplemented with 0.01% Tween 20 and various concentrations (10, 20, 40, 60, 80, and 100 mg/L) of DEHP at 160 rpm and 28°C for 48 h. Diluted NB supplemented with 0.01% (v/v) of Tween 20 and 0.5% (v/v) of 80% acetone was used as control. After incubation, cell cultures were serially diluted in 0.85% saline solution and incubated onto diluted NA. After incubation at 28°C for 48 h, bacterial CFU were counted. The experiment was conducted twice with five replicates each. For further study, *A. citrulli* was cultured in diluted NB (1:10) supplemented with 0.01% Tween 20 and DEHP at a final concentration of 20 mg/L based on the amount remaining in the soil (Liu et al., 2019) at 160 rpm and 28°C for 48 h.

### Lipid peroxidation assay

Bacterial lipid peroxidation after DEHP exposure was measured using a lipid hydroperoxide assay kit according to the manufacturer’s instructions (Cayman Chemical Co., MI, USA). *A. citrulli* suspension was prepared as described above; the cells were harvested by centrifugation at 13,000 rpm for 10 min at 4°C and used for the lipid peroxidation assay. The absorbance was measured at 500 nm using a microplate reader (Infinite M200 Pro, Tecan, Switzerland). The experiment was conducted twice with ten replicates each.

### Membrane permeability

Bacterial membrane permeability assay was performed according to the method described by Loh et al. (1984). The bacterial cells were prepared as described above; cell pellets were resuspended in 5 mM HEPES buffer (pH 7.2), and the concentration was adjusted to an optical density (OD)_600_ of 0.5. Further, 50 µL of 1-N-phenylnaphthylamine (NPN; 40 µM stock) and 50 µL of 5 mM HEPES buffer (pH 7.2) containing 5 mM glucose and 1 mM sodium azide were added to 100 µL of bacterial suspension (A_1_). A solution containing 50 µL of NPN (40 μM stock) and 150 µL of 5 mM HEPES buffer (pH 7.2) (A_0_) was used as background. The fluorescence was immediately measured using Hidex Sense microplate reader (Hidex, Finland) at an excitation and emission wavelength of 355 nm and 405 nm, respectively; slit width was set to 5 nm. The experiment was conducted twice with ten replicates each.


$$\text{Membrane permeability}(\text{relative fluorescence unit}\left(\text{RFU}\right))={\text{A}}_{1}/{\text{A}}_{0}$$

### Nucleic acid leakage

The bacterial cells were harvested in the logarithmic phase by centrifugation at 4,000 rpm for 10 min, washed twice with 0.85% saline solution, resuspended in 0.85% saline solution, and added to 20 mg/L of DEHP at 160 rpm and 28°C for 3 h. The samples were harvested by centrifugation at 4,000 rpm for 10 min, and absorbance of the supernatant was measured at 260 nm. The experiment was conducted twice with three replicates each (Fu et al., 2008).

### Fatty acid methyl esters

The bacterial cells were harvested by centrifugation at 13,000 rpm for 10 min, and the sample and internal standard (pentadecanoic acid; Sigma, USA) were placed in tubes with teflon-lined caps. A methylation mixture containing methanol: benzene: 2,2-dimethoxy-propane (DMP): H_2_SO_4_ (39: 20: 5: 2) was used, and a mixture of heptane was added to the sample. Afterwards, the tube was placed in a water bath at 80°C. The supernatants were determined by gas chromatography (Agilent 7890A, Agilent Technologies, USA) equipped with a column (DB-23, 120 mm × 0.25 mm × 0.25 μm) and a flame ionization detector. The temperature of the injector and detector was set at 250°C and 280°C, respectively. The experiment was conducted in triplicate (Garcés and Mancha, 1993).

### ATPase activity

ATPase activity assay was performed using an ATPase/GTPase activity assay kit according to the manufacturer’s instructions (MAK113, Sigma). *A. citrulli* was prepared as described above; the cells were harvested by centrifugation at 13,000 rpm for 10 min at 4°C, and the pellets were suspended in assay buffer [40 mM Tris buffer (pH 7.5), 80 mM NaCl, 8 mM MgAc_2_, 1 mM EDTA]. Ten µL of 4 mM ATP was added to 20 µL of cell suspension, and the cells were incubated for 30 min at 25°C. Subsequently, 200 µL of reagent was added to each well, and the samples were incubated for another 30 min at room temperature. Their absorbance was measured at 620 nm using a microplate reader. The experiment was conducted twice with five replicates each.

### Tricarboxylic acid cycle

The bacterial cells were cultured and harvested as described above. Quantitative analyses of citrate, glutamate, oxaloacetate, pyruvate, isocitrate, malate, fumarate, succinate, and alpha-ketoglutarate (α-KG) were performed using citrate (MAK057, Sigma), glutamate (MAK004, Sigma), oxaloacetate (MAK070, Sigma), pyruvate (MAK071, Sigma), isocitrate (MAK061, Sigma), malate (MAK067, Sigma), fumarate (MAK060, Sigma), succinate colorimetric (MAK184, Sigma), and α-KG (MAK054, Sigma) assay kit, respectively. Succinate dehydrogenase activity was analyzed using a succinate dehydrogenase assay kit (MAK197; Sigma-Aldrich). The experiment was conducted twice with five replicates each.

### RNA sequencing and quantitative real-time polymerase chain reaction

*A. citrulli* was prepared as described above; the cell pellets were harvested by centrifugation at 13,000 rpm for 10 min at 4°C. For RNA extraction, total RNA was extracted by using Total RNA extraction kit (iNtRON, South Korea). After quantification using Nanodrop (ND-1000, Thermo Scientific, USA), total RNA was analyzed by Macrogen Inc. (South Korea) for RNA-seq. Differentially expressed genes (DEGs) between DEHP-treated and untreated cells were analyzed using the DESeq2 R library. DEG gene lists were independently submitted for functional annotation using the Database for Annotation Visualization and Integrated Discovery (DAVID). Each sample was assayed in triplicate. Raw reads were deposited in the Sequence Read Archive (SRA) database of NCBI under BioProject accession number PRJNA1002002. For quantitative real-time PCR validation of RNA-seq, 0.6 μg of total RNA was synthesized to complementary DNA using TOPscript RT DryMIX (Enzynomics, South Korea), and a CFX96 Real-time PCR Detection system (Bio-Rad, USA) and primers were used (**Supplementary Table S1**). The real-time PCR was conducted as follows: 95°C for 10 min; 40 cycles of 95°C for 15 s, 58°C for 20 s, and 72°C for 18 s. As reference primer, *rpoB* was used. For relative expression of pathogenicity-related genes using qRT-PCR, the synthesized cDNA was used, and relative quantities of specific transcripts were determined using SYBR Green RT-PCR reagents. The primers used for qRT-PCR are listed in **Table 1**, and the qRT-PCR was performed as follows: 95°C for 10 min, followed by 40 cycles at 95°C for 15 s, 56°C for 30 s, and 72°C for 30 s. The relative expression of genes of interest was calculated using the 2^-△△CT^ method (Livak and Schmittgen, 2001). The experiment was conducted twice with five replicates each.

**Table 1:** Primers used in this study for evaluating relative gene expression of virulence genes of *Acidovorax citrulli*.

Primer	Sequence (5’-3’)	Function	Reference
*hrpX*-F	5’-GGGAGGCATTCAAGCCATCT-3’	T3SS-related genes	Zhao et al., (2020)
*hrpX*-R	5’-AACAGCAGCCAGGCGAGTT-3’		
*hrpG*-F	5’-GTCGCACCCTGCTGCTGATAG-3’	T3SS-related genes	Zhao et al., (2020)
*hrpG*-R	5’-AGGCGTGGTCCGACAGTTCTT-3’		
*rpoB*-F	5’-GCGACAGCGTGCTCAAAGTG-3’	Reference gene	Zhao et al., (2020)
*rpoB*-R	5’-GCCTTCGTTGGTGCGTTTCT-3’		

### Swimming motility assay and biofilm formation

Swimming motility assay was performed on NB containing 0.2% agar (SHOWA, Japan) using the method described by Rashid and Kornberg (2000) with slight modification. The bacterial cells were prepared as described above, suspended in 0.85% NaCl, and 2 µL of bacterial suspension (10^4^ CFU/mL) was inoculated into NB. The cells were incubated at 28°C, and the colony diameter was measured for consecutive three days after inoculation. The experiment was conducted twice with five replicates each.

For biofilm formation assay, the bacterial cell pellets, prepared as described above, were adjusted to 10^4^ CFU/mL by dilution with NB. The cell suspension (130 µL) was added into a 96-well polyvinyl chloride plate and incubated (Falcon 353911, Corning, USA) at 28°C for three days; subsequently, the bacterial suspension was removed and washed three times with sterile distilled water. Crystal violet (0.1%) was added to the wells for staining the cells, which were then incubated for 30 min at room temperature, and then washed with sterile distilled water. The stained cells were suspended in 95% ethanol for 30 min, and their absorbance was measured at 590 nm using a microplate reader (Coffey and Anderson, 2014). The experiment was conducted twice with five replicates each; each replicate was performed in five wells.

### Statistical analyses

Data analyses, including the calculation of average values, standard errors, analysis of variance (ANOVA), and least significant difference (LSD) test at *P* < 0.05, were performed using R (version 1.4.1106, USA).

## Results

### Effect of di-(2-ethylhexyl) phthalate on the bacterial population and membrane of *Acidovorax citrulli*

**Figure 1:**
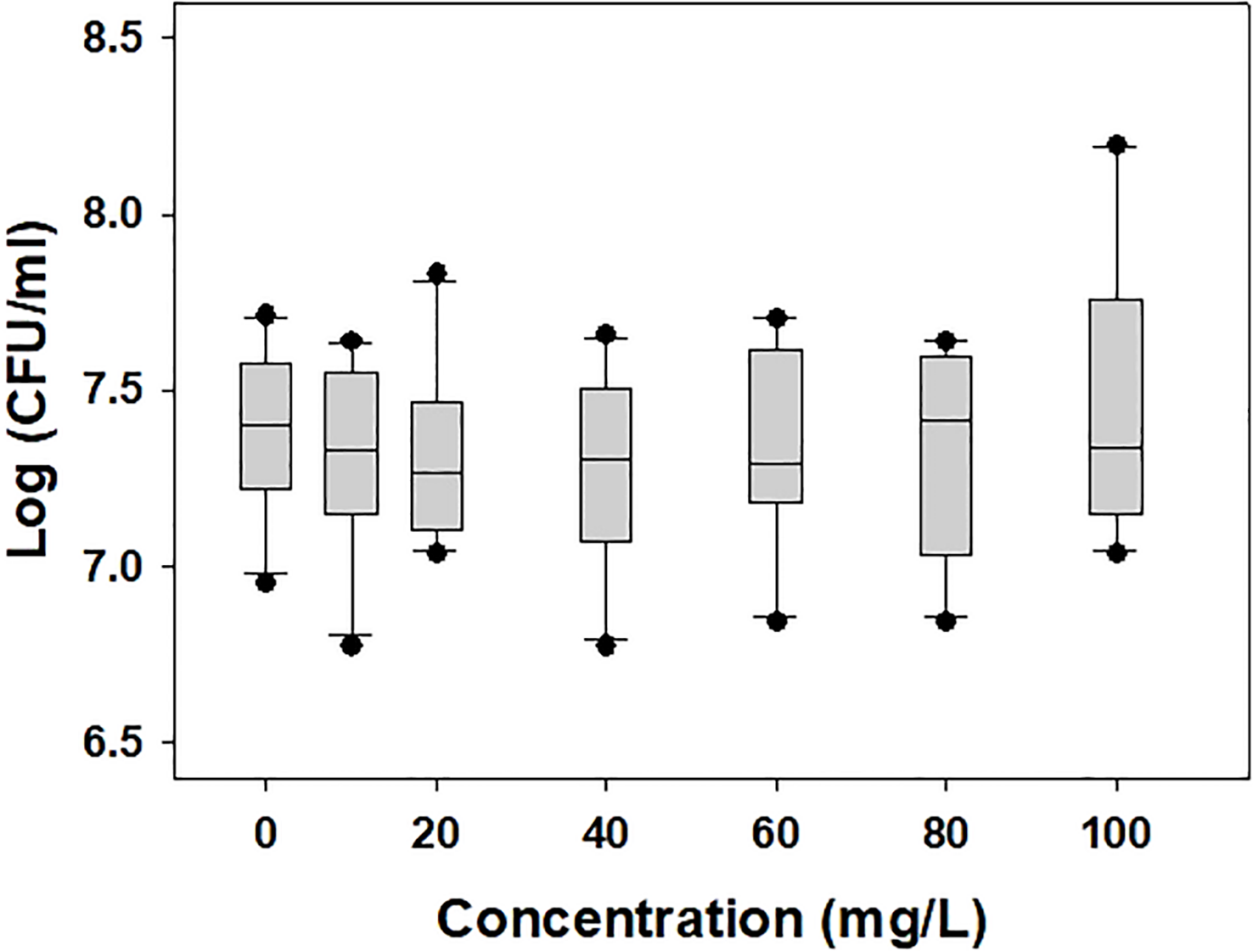
Bacterial population of di-(2-ethylhexyl) phthalate-exposed *Acidovorax citrulli.* No statistically significant differences were observed in LSD test (*P* < 0.05).

The population of *A. citrulli* was not affected by various concentrations (0, 10, 20, 40, 60, 80, and 100 mg/L) of DEHP present in the NB (**Figure 1**). To examine the effects of DEHP exposure on the membrane of *A. citrulli*, malondialdehyde (MDA) content, membrane permeability, and nucleic acid leakage were evaluated (**Table 2**). DEHP treatment tended to increase lipid peroxidation, membrane permeability, and nucleic acid leakage, however, significant differences were not observed (**Table 2**). The membrane of *A. citrulli* is mainly comprised of saturated fatty acids, including C12:0 (lauric acid), C14:0 (myristic acid), C16:0 (palmitic acid), C17:0 (margaric acid), and C18:0 (stearic acid), and unsaturated fatty acids, including C16:1 (palmitoleic acid) and C18:1n9c (oleic acid); the amount of fatty acids in the membrane of DEHP-exposed *A. citrulli* increased compared to that of unexposed, and therefore, the ratio of fatty acid content of exposed and unexposed cells was more than 1.0 (**Table 3**). Notably, the content of C16:0 and C16:1 were increased, whereas significant increase was observed in only that of C16:1 in response to DEHP exposure (**Table 3**).

**Table 2:** Lipid peroxidation, membrane permeability, and nucleic acid leakage in di(2-ethylhexyl) phthalate-exposed and unexposed *Acidovorax citrulli*.

Treatment	Lipid peroxidation	Membrane permeability	Nucleic acid leakage
	(μM)	Relative fluorescence unit (RFU)	Optical density at 260 nm (OD_260_)
Unexposed	22.8 ± 3.8^a^	461.8 ± 4.6	0.0096 ± 0.0031
Exposed	25.8 ± 3.5	472.8 ± 6.8	0.0178 ± 0.0044

a Values indicate mean ± standard error, and no statistically significant difference by LSD at P < 0.05.

**Table 3:** Fatty acid methyl ester (FAME) composition of di(2-ethylhexyl) phthalate-exposed and unexposed *Acidovorax citrulli*.

Fatty acid methyl ester	Amount (mg/g)		Fold (Exposed/Unexposed)
	Unexposed	Exposed	
Lauric acid (dodecanoic acid, C12:0)	1.102 ± 0.022^a^	1.113 ± 0.024	1.01
Myristic acid (tetradecanoic acid, C14:0)	0.784 ± 0.004	0.804 ± 0.013	1.02
Palmitic acid (hexadecanoic acid, C16:0)	15.115 ± 0.145	15.455 ± 0.190	1.02
Palmitoleic acid (C16:1)	16.090 ± 0.240	17.280 ± 0.260*	1.07
Margaric acid (heptadecanoic acid, C17:0)	0.401 ± 0.008	0.412 ± 0.005	1.03
Stearic acid (octadecanoic acid, C18:0)	0.197 ± 0.004	0.199 ± 0.003	1.01
Oleic acid (C18:1n9c)	0.180 ± 0.007	0.180 ± 0.002	1.00

a Values indicate mean ± standard error, and an asterisk means statistical difference by LSD at P < 0.05.

### Influence of di-(2-ethylhexyl) phthalate on metabolism of *Acidovorax citrulli*

**Figure 2:**
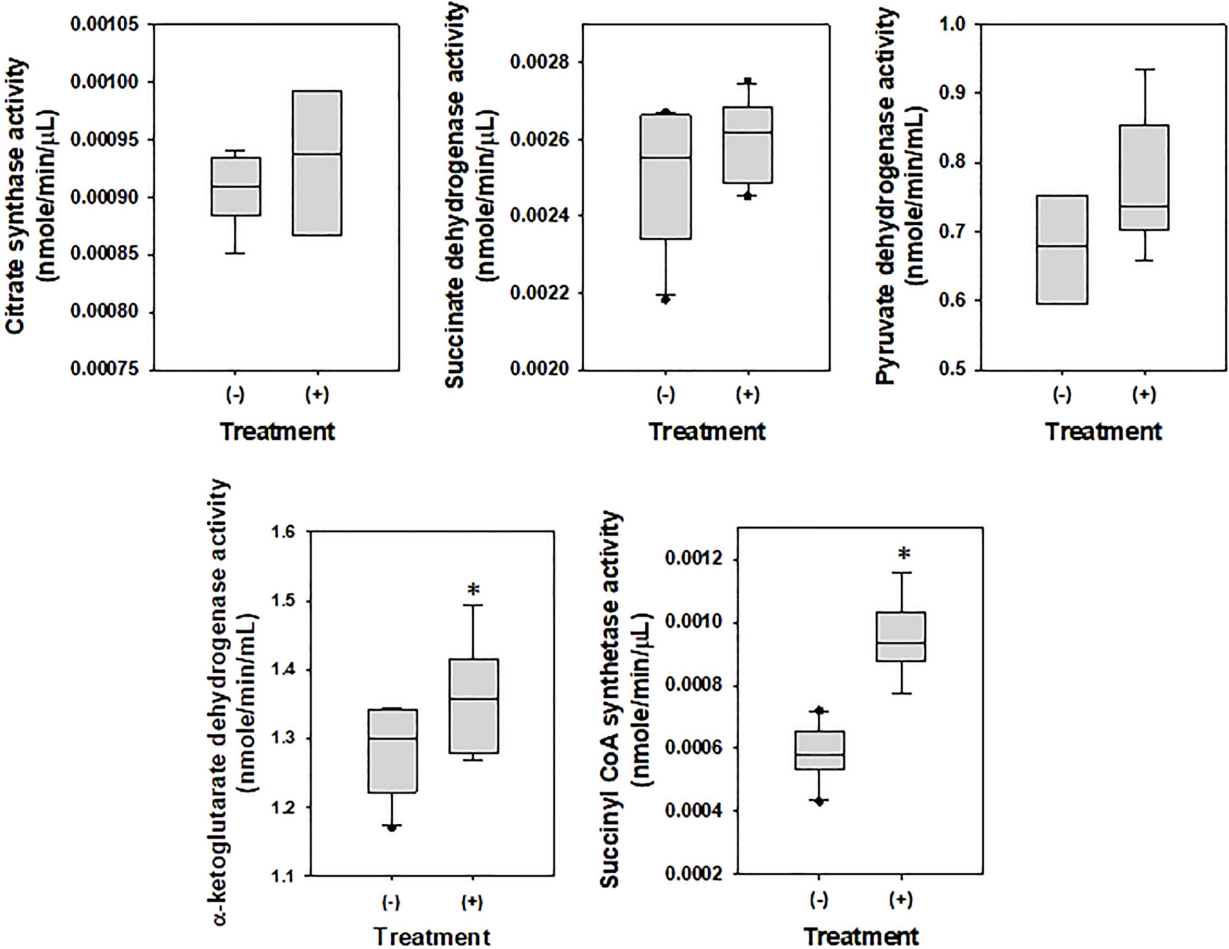
Effects of di-(2-ethylhexyl) phthalate on enzymes related to glycolysis and TCA cycle. (-) indicates DEHP-unexposed *Acidovorax citrulli*, and (+) indicates DEHP-exposed *A. citrulli*. An asterisk indicates a statistically significant difference as determined by LSD test (*P* < 0.05).

**Figure 3:**
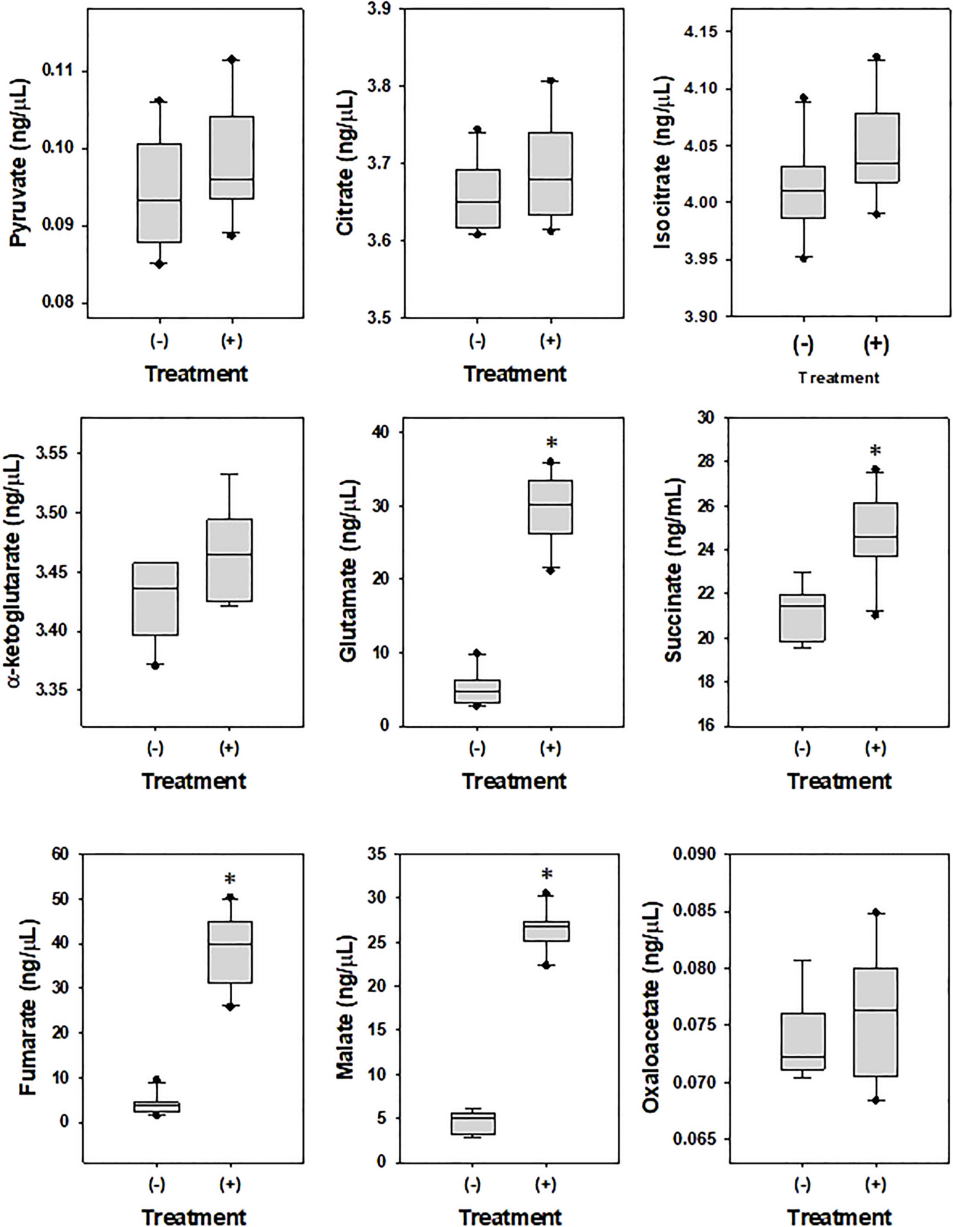
Effects of di-(2-ethylhexyl) phthalate (DEHP) on metabolites in TCA cycle. (-) indicates DEHP-unexposed *Acidovorax citrulli*, and (+) indicates DEHP-exposed *A. citrulli*. An asterisk indicates a statistically significant difference as determined by LSD test (*P* < 0.05).

To examine the effects of DEHP on the metabolism of *A. citrulli*, ATPase (**Table 4**) and TCA cycle-related components and enzymes were investigated (**Figures 2**, **3**). DEHP treatment did not influence ATPase activity; however, the production of glutamate, malate, succinate, and fumarate in the TCA cycle of *A. citrulli* was significantly increased compared to that in the unexposed control (**Figure 2**). As a result, TCA cycle-related enzyme activity, including α-ketoglutarate dehydrogenase and succinyl-CoA synthetase, in DEHP-exposed *A. citrulli* was effectively increased compared to that in the unexposed control (**Figure 3**).

**Table 4:** ATPase activity of di(2-ethylhexyl) phthalate-exposed and unexposed *Acidovorax citrulli*.

Treatment	ATPase activity (U/L)
Unexposed	9.68 ± 0.62^a^
Exposed	10.15 ± 0.47

a Values indicate mean ± standard error, and no statistically significant difference by LSD at P < 0.05.

### Transcriptome analysis using RNA sequencing

**Figure 4:**
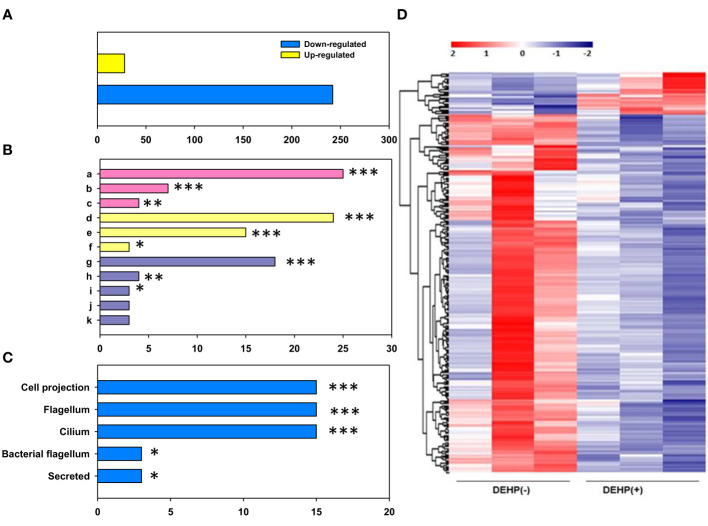
RNA sequencing of *Acidovorax citrulli*. **(A)** Significant count by fold change and p-value; Number of upregulated and downregulated genes. **(B)** Gene ontology categories: Biological Process (BP, pink), Molecular Function (MF, yellow), and Cellular Component (CC, purple); a: translation, b: bacterial-type flagellum-dependent cell motility, c: bacterial-type flagellum assembly, d: structural constituent of ribosome, e: rRNA binding, f: structural molecule activity, g: ribosome, h: extracellular region, i: bacterial-type flagellum hook, j: small ribosomal subunit, k: large ribosomal subunit. **(C)** Functional annotation. **(D)** Heat map of the expression patterns of di-(2-ethylhexyl) phthalate (DEHP)-exposed and unexposed *A. citrulli*; Red and blue colors represent the maximum and minimum values, respectively. **(B, C)**; DEHP-exposed *A. citrulli* showed a significant decrease in differentially expressed genes compared to the unexposed control. *, **, and *** indicate statistically significant differences at P < 0.05, P < 0.01, and P < 0.001, respectively.

The gene expression of DEHP-exposed *A. citrulli* was compared with that of the unexposed control using transcriptome and gene ontology (GO) analyses. RNA-seq results showed that 270 genes were differentially expressed in DEHP-exposed *A. citrulli* compared to the unexposed control; 28 genes were upregulated and 242 genes were downregulated (**Figure 4**). GO analysis divided DEGs into genes related to biological processes (BP), molecular functions (MF), and cellular components (CC); among BP, genes related to translation (GO:0006412), flagellum-dependent cell motility (GO:0071973), and flagellum assembly (GO:0044780) were significantly downregulated (**Figure 4**). In addition, DAVID analysis confirmed that the genes downregulated by DEHP treatment were mainly associated with cellular components (bacterial flagellum and cilium) (**Figure 4**). Among upregulated 28 genes by DEHP-exposure, 14 genes were hypothetical protein, and the others belonged to various categories such as ATP-binding cassette domain-containing protein, ABC transporter ATP-binding protein, the others were belonging to SDR family NAD(P)-dependent oxidoreductase, outer membrane beta-barrel protein and so on. The similar result to RNA-seq data were validated by qRT-PCR in **Supplementary Table S1**. Overall, most DEGs associated with translation, the flagellum assembly and flagellum-dependent motility were downregulated in DEHP-exposed *A. citrulli*, which may lead to a loss of motility.

### Impact of di-(2-ethylhexyl) phthalate on virulence traits of *Acidovorax citrulli in vitro*

**Figure 5:**
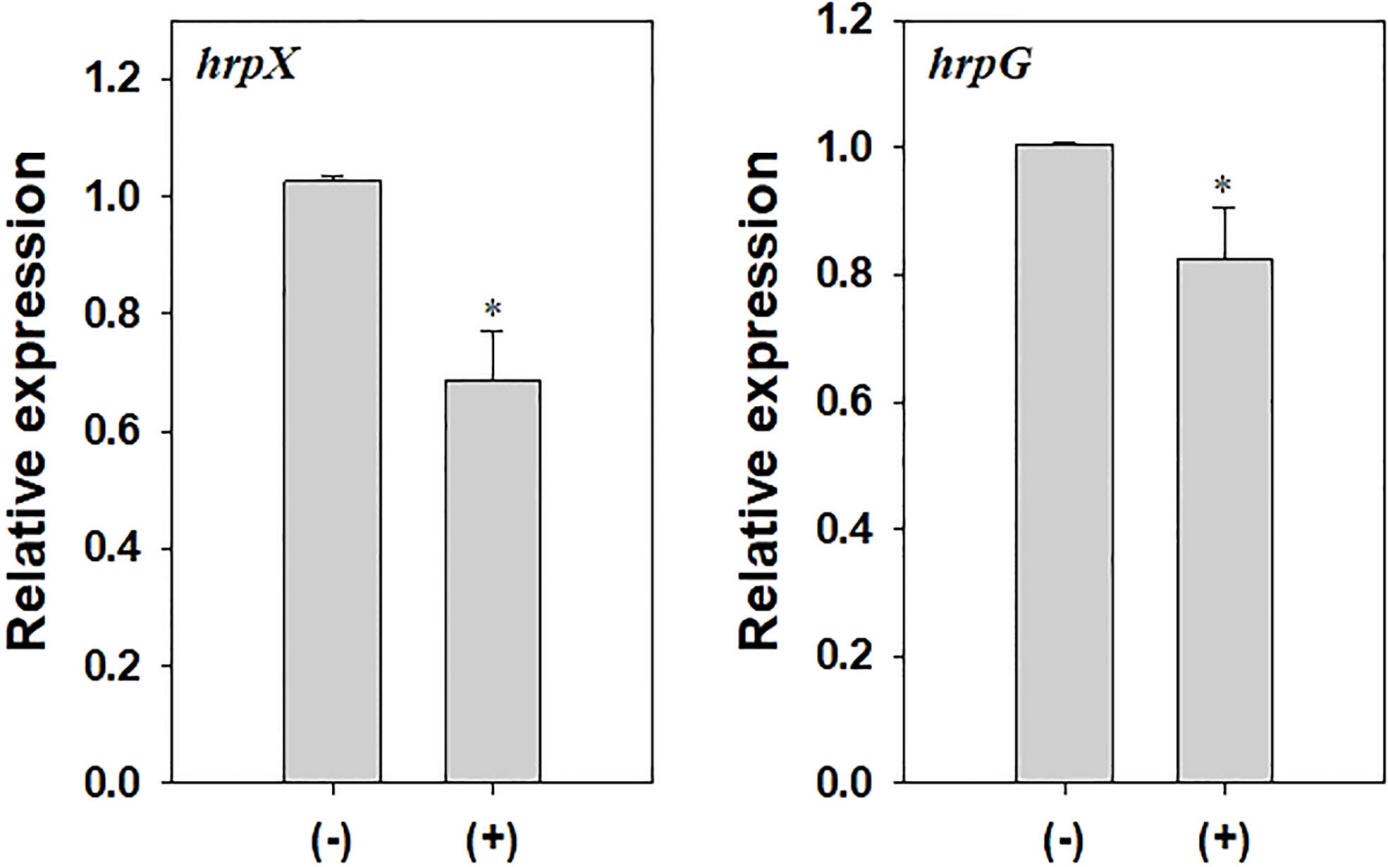
Relative expression of *hrpX* and *hrpG* in *Acidovorax citrulli*. (-) indicates di-(2-ethylhexyl) phthalate (DEHP)-unexposed *A. citrulli*, and (+) indicates DEHP-exposed *A. citrulli*. An asterisk indicates a statistically significant difference as indicated by LSD test (*P* < 0.05); error bars indicate standard error.

To investigate changes in the virulence of *A. citrulli* caused by DEHP exposure, pathogenicity-related gene expression and virulence traits, including swimming motility and biofilm formation, were tested. The relative gene expression of *hrpX* and *hrpG* in DEHP-exposed *A. citrulli* was significantly decreased by 0.67- and 0.82-folds, respectively, compared to that in the unexposed control (**Figure 5**). In addition, DEHP treatment significantly decreased the swimming motility of *A. citrulli*; however, biofilm formation was not affected (**Table 5**).

**Table 5:** Swimming motility and biofilm formation of di(2-ethylhexyl) phthalate-exposed or unexposed *Acidovorax citrulli*.

Treatment	Swimming motility	Biofilm formation
	Halo diameter (mm)	Absorbance at 590 nm
Unexposed	66.40 ± 0.60*^a^	0.24 ± 0.006
Exposed	60.20 ± 1.37	0.22 ± 0.006

a Values indicate mean ± standard error, and an asterisk means statistical difference by LSD at P < 0.05.

## Discussion

In this study, we evaluated the effects of DEHP on bacterial population and metabolism of *A. citrulli* and the resulting changes in its virulence traits. Pollutants have been known to affect bacterial cell structure, including permeability and membrane stability, and physiological activity (Qian et al., 2016; Chen et al., 2018). Wang et al. (2019a) reported that the treatment with 20, 40, and 80 mg/L of dimethyl phthalate inhibited the growth of *Escherichia coli* along with severe cell membrane disruption and increased oxidative stress. In the present study, 20 mg/L DEHP treatment did not affect the population of *A. citrulli in vitro*; however, it tended to increase lipid peroxidation, membrane permeability, and nucleic acid leakage. The results indicated the absence of adverse effects on the survival of *A. citrulli* treated with all tested concentrations of DEHP. Similarly, Kim et al. (2019) used DEHP solution (10–20,000 mg in 1 kg of dried soil) for evaluation of soil ecotoxicity through assay on plant, earthworm, soil algae, Collembola, and nematodes; they observed low toxicity at only high concentrations of DEHP, and no effect on soil eukaryotic species at environmentally relevant concentrations. Liu et al. (2019) reported that the amount of six phthalic acid esters, including benzyl butyl phthalate, dubutyl phthalate, di-(2-ethylhexyl) phthalate, diethyl phthalate, dimethyl phthalate, and di-n-octyl phthalate, ranged from 1.2 to 7.3 mg/kg in vegetable fields; in particular, high concentration of DEHP was detected (0.48–15.34 mg/kg) in cultivated soils of China. The diverse range of accumulated amounts might be dependent on soil characteristics, samples, regions, and types of phthalate. Thus, the 20 mg/L of DEHP used in this study was assumed as highly relevant concentration in soils, the concentration of DEHP could be less influence on survival of *A. citrulli*.

However, DEHP caused changes in the FAME membrane composition of *A. citrulli*. Exposure to pollutants leads to change in the permeability and hydrophobicity of bacterial cell membranes (Al-Tahhan et al., 2000; Wang et al., 2019b), and remodeling of membrane lipid composition is essential to adapt in response to environmental changes (Čertík et al., 2003). Chlorophenol, a soil and water contaminant, affected the growth, lipid content, and fatty acid composition of the membrane of the bacterium *Kocuria varians*. In particular, it markedly enhanced the accumulation of palmitoleic and oleic acids; however, the content of phosphatidylcholine and phosphatidylethanolamine, two main unsaturated lipids, decreased (Dercová et al., 2004). The current study findings indicate that the presence of DEHP caused changes in fatty acid composition of *A. citrulli*, and that palmitoleic acid (C16:1) was mainly sensitive to DEHP.

During analysis of TCA cycle-related components and enzymes, DEHP-exposed *A. citrulli* showed increased production of glutamate, malate, succinate, and fumarate and induced activation of α-ketoglutarate dehydrogenase and succinyl-CoA synthetase in TCA cycle. TCA cycle, also known as the citric acid cycle, is a metabolic pathway that connects carbohydrate, fat, and protein metabolism and plays a major role in energy production. Fumarate (Zheng et al., 2015), succinate (Dalla Pozza et al., 2020), α-ketoglutarate dehydrogenase (Kehinde et al., 2022), and succinyl-CoA synthetase (Elpeleg et al., 2005) play important roles in TCA cycle. TCA cycle is generally activated during biodegradation of organic pollutant (Seo et al., 2013); for example, succinate accumulation was observed in *Pseudomonas* sp. strain HF-1 during nicotine degradation (Ye et al., 2012) and in *Rhodococcus* sp. YYL during tetrahydrofuran degradation (He et al., 2014). *A. citrulli* significantly accumulated succinate, fumarate, and malate, which are serially degraded in TCA cycle, and activated α-ketoglutarate dehydrogenase and succinyl-CoA synthetase in response to DEHP treatment.

Bacterial flagella are involved in motility, adherence, colonization, biofilm formation, and virulence (Bahar et al., 2010; Soria-Bustos et al., 2022). The flagellum is primarily composed of basal body rings and tubular axial structures (hooks, filaments, and rods), which produce thrust for the cells to swim in viscous environment and cause smooth transmission of motor torque to the filament regardless of its orientation (Imada, 2018). The flagellar motor switch protein (Flig) is one of the three proteins encoded by *filM* in certain bacteria. The protein complex regulates the direction of flagellar rotation and hence controls swimming behavior. The switch protein is a complex protein that responds to signals transduced by chemotactic sensory signaling system during chemotactic behavior (Roman et al., 1993). In this study, all DEGs involved in flagellar assembly, including *fliM*, *fliS*, *fliD*, *flhA*, *flgA*, and *flgG*, were downregulated in DEHP-treated *A. citrulli.* This suggests that DEHP exposure reduced flagellar assembly. In contrast, 28 proteins were upregulated in DEHP-treated cells compared to that of the untreated control, 14 of which were hypothetical proteins; elevated expression was observed for one protein associated with sugar transporter system, ABC transporter protein, and ATP-binding protein. ABC transporters present in bacteria take up various nutrients, vitamins, and ions and secrete toxic compounds (Moussatova et al., 2008). Pollutants alter the microbial community and increase the abundance of genes encoding ABC transporters and other proteins (Xu et al., 2018; Chen et al., 2020). Similarly, ABC transporter and ATP-binding protein gene expression was likely increased in response to toxin release by DEHP.

Biofilm formation and swimming motility are important virulence factors of *A. citrulli*. Our study revealed that the swimming motility of *A. citrulli* was decreased by DEHP exposure, whereas no difference was observed in biofilm formation. Many plant pathogenic bacteria secrete protein effectors into the host cells through T3SS (Buttner and He, 2009), which causes disease in susceptible plants and induces hypersensitivity reactions in resistant plants. T3SS is encoded by *hrp* (hypersensitive reaction and pathogenicity) genes. *hrpG* is a key regulatory gene of T3SS in *A. citrulli* and an OmpR-type regulator that can activate the transcription of the AraC-type activator *hrpX* (Zhang et al., 2018). Deletion of *hrpG* and *hrpX* in *A. citrulli* reduced pathogenicity in watermelon seedlings. We showed that *hrpX* and *hrpG* gene expression of *A. citrulli* was decreased after DEHP treatment. These results suggest that DEHP could affect the virulence factors of *A. citrulli* in laboratory conditions.

Our study revealed that exposure to DEHP in tested concentration did not significantly affect the survival of *A. citrulli*; however, it led to altered fatty acid composition of the bacterial membrane. DEHP exposure increased the production of certain metabolites in TCA cycle and activated TCA-related enzymes. Additionally, DEHP exposure reduced gene expression related to flagellar assembly, and increased the expression of genes encoding ABC transporter and ATP-binding proteins, which are involved in the uptake of nutrients and secretion of toxic compounds. Moreover, it influenced virulence traits, including swimming activity, and expression of pathogenicity genes, including *hrpX* and *hrpG*. Taken together, these results suggest that accumulation or release of DEHP in soil environment could potentially affect the metabolism and virulence traits of *A. citrulli*.

## Data availability statement

The original contributions presented in the study are included in the article/**Supplementary Materials**. Further inquiries can be directed to the corresponding author.

## Author contributions

Y-RK: investigation, writing-original draft preparation, and analysis. MS: conceptualization, data curation, analysis, and writing-review & editing. All authors contributed to the article and approved the submitted version
