# Supplementary material for: Effects of di-(2-ethylhexyl) phthalate on growth, metabolism, and virulence of the plant pathogenic bacterium Acidovorax citrulli
Source: Front Cell Infect Microbiol. 2023 Aug 25;13:1228713. doi: 10.3389/fcimb.2023.1228713 (PMC10485622; doi:10.3389/fcimb.2023.1228713)
Supplement: Supplementary file 1 [file Table_1.docx]

Supplementary Table

Table S1. Primers used for quantitative Real-Time PCR validation of RNA-seq result

| Gene ID | Primer  name | Gene name | Forward (5’-3’) | Reverse (5’-3’) | RNA-seq^a^ | qPCR^b^ |
| --- | --- | --- | --- | --- | --- | --- |
| Reference gene | rpoB |  | GCGACAGCGTGCTCAAAGTG | GCCTTCGTTGGTGCGTTTCT |  |  |
| 69632220 | flhD | flagellar transcriptional regulator FlhD | CCTCGGAACAACTTCTCGCT | AGAGGGAGGCCAGGATGTC | - 3.15 | - 0.05 |
| 69632221 | flhC | flagellar transcriptional regulator FlhC | TACAAGGAAATCGCCGGCAA | GTAGGCCTTGGTGAGCAGTT | - 3.05 | - 0.22 |
| 69632918 | ACD | ATP-binding cassette domain-containing protein | CACCGAGACGATGGAGAACG | GCTGCCTTCTCCGTTTCCTT | 2.37 | 0.49 |
| 69633263 | fliM | flagellar motor switch protein FliM | GAAGAAGTCGATGCCCTGCT | GATTTCCATCGTCGGCATGC | - 2.35 | - 0.42 |
| 69633273 | fliS | flagellar export chaperone FliS | CTGGAGGAAGGGTTGAGCAC | CTTCTTCCAGCATTGCGTCG | - 2.70 | - 0.92 |
| 69633274 | fliD | flagellar filament capping protein FliD | GGCAAGGACCAGTCGTACAA | TGTTGAGCGCGTTCATGTTG | - 2.41 | - 0.21 |
| 69633288 | flhA | flagellar biosynthesis protein FlhA | GAAGGAAGCCGCACAAATGG | CTTCCTCGATGAGCTTGGGG | - 2.33 | 0.32 |
| 69633295 | flgB | flagellar basal body rod protein FlgB | TGCTCGACAAGTTGACCGAA | GAAATTGAAGTCGCGCGCC | - 2.41 | - 0.59 |
| 69633296 | flgC | flagellar basal body rod protein FlgC | GTGCTCGATCCCACCCATC | CGACTTGGTGGTGTTGAGGA | - 2.60 | - 0.83 |
| 69633298 | flgE | flagellar hook protein FlgE | TCGGCCAGATCAAGTTCGAC | TCGAGCTTGATGTTCACCGT | - 2.97 | - 0.35 |
| 69633299 | flgF | flagellar basal-body rod protein FlgF | GATACCCAACGACCCGAACG | TGTCTTGTCGTTGGTCTCGG | - 2.33 | 0.15 |
| 69633300 | flgG | flagellar basal-body rod protein FlgG | GCATGGCGAGCTTCATCAAC | CAGCTCTTCCACCACGTTCA | - 3.18 | - 0.54 |
| 69633304 | flgJ | flagellar assembly peptidoglycan hydrolase FlgJ | GGAGATCACCACCACCGAAT | CTTCTCGTAGCGTGGGTTGT | - 2.74 | 0.10 |
| 69633305 | flgK | flagellar hook-associated protein FlgK | ACCGACATGCTCAACTCGTT | TGAGCTGTTCCTTCACGGTG | - 2.08 | - 0.83 |
| 69632472 | T6 | type IV pilin protein | TCCTTCACCGTGCCTTTCAA | GAGAGATCACCCACGGCAG | - 2.55 | 0.07 |
| 69632473 | PilY | pilus assembly protein PilY | TGATCCCGCCAACAATGTCA | AAGGAACGCTCGTATTGGCA | - 2.42 | - 0.35 |
| 69632474 | PUA | pilus assembly protein | TCGTCCTGCTGATCCTGGTA | TCCAGTTCCGCATCGATGAG | - 2.46 | - 0.10 |
| 69633643 | ABC1 | ABC transporter ATP-binding protein | GTGGCCATCCTGCTCGTG | GTATCGGTCCTTGGTCGCTT | 3.11 | 1.07 |
| 69635590 | ABC2 | ABC transporter ATP-binding protein | AAGACGACGCTGATCCACC | GCTGGTGATCTGGTAGGAGC | 2.39 | 0.99 |
| 69633748 | SDR | SDR family NAD(P)-dependent oxidoreductase | CGCCAGTCCAACTACGTCTA | TTGAGGTGCGCGGTCATC | 2.38 | 1.02 |
| 69633774 | Tae4 | T6SS effector amidase Tae4 family protein | TGAAGCGCATTGAAAGCCG | CCAAACGGCGTATGTACACTG | - 2.87 | - 0.39 |

^a^ Mean log2fold change value of three replicates, ^b^ -ΔΔCt value of qPCR
